# Elucidation of the Dual Role of Mycobacterial MoeZR in Molybdenum Cofactor Biosynthesis and Cysteine Biosynthesis

**DOI:** 10.1371/journal.pone.0028170

**Published:** 2011-11-30

**Authors:** Martin Voss, Manfred Nimtz, Silke Leimkühler

**Affiliations:** 1 Institute of Biochemistry and Biology, University of Potsdam, Potsdam, Germany; 2 Helmholtz Center for Infection Research, Braunschweig, Germany; National Institute for Medical Research, Medical Research Council, United Kingdom

## Abstract

The pathway of molybdenum cofactor biosynthesis has been studied in detail by using proteins from *Mycobacterium* species, which contain several homologs associated with the first steps of Moco biosynthesis. While all *Mycobacteria* species contain a MoeZR, only some strains have acquired an additional homolog, MoeBR, by horizontal gene transfer. The role of MoeBR and MoeZR was studied in detail for the interaction with the two MoaD-homologs involved in Moco biosynthesis, MoaD1 and MoaD2, in addition to the CysO protein involved in cysteine biosynthesis. We show that both proteins have a role in Moco biosynthesis, while only MoeZR, but not MoeBR, has an additional role in cysteine biosynthesis. MoeZR and MoeBR were able to complement an *E. coli moeB* mutant strain, but only in conjunction with the *Mycobacterial* MoaD1 or MoaD2 proteins. Both proteins were able to sulfurate MoaD1 and MoaD2 *in vivo*, while only MoeZR additionally transferred the sulfur to CysO. Our *in vivo* studies show that *Mycobacteria* have acquired several homologs to maintain Moco biosynthesis. MoeZR has a dual role in Moco- and cysteine biosynthesis and is involved in the sulfuration of MoaD and CysO, whereas MoeBR only has a role in Moco biosynthesis, which is not an essential function for *Mycobacteria*.

## Introduction

Among the metabolic pathways requiring sulfur transfer are those leading to the formation of FeS clusters, biotin, thiamin, lipoic acid, molybdopterin (MPT), and sulfur-containing bases in RNA [1]. In addition, a new pathway for cysteine biosynthesis involving sulfur transfer has been elucidated in *Mycobacterium tuberculosis* recently [2], [3]. This pathway involves the ubiquitin-fold protein CysO that contains a C-terminal thiocarboxylate group at the last glycine of a GG-motif as sulfide donor [4]. Similar proteins involved in sulfur transfer are the ThiS protein involved in thiamin biosynthesis [5] and the MoaD protein involved in molybdopterin biosynthesis [6]. The *M. tuberculosis* protein MoeZR has been shown to transfer sulfur onto CysO for the formation of the thiocarboxylate group. The cysteine synthase M (CysM) catalyzes the addition of *O*-phosphoserine to the carboxy-terminus of the protein-bound CysO-thiocarboxylate to generate a cysteine-CysO adduct [7]. The protease Mec^+^ hydrolyzes the CysO-cysteine adduct to release cysteine and regenerate CysO [2]. The CysO activating enzyme MoeZ belongs to a superfamily of proteins consisting of related proteins that are members of pathways involved in the transfer of sulfur-containing moieties to metabolites [8]. Members of this family are referred to as MoeB, MoeBR, MoeZR, MoeZ and MOCS3 [9]. MoeB, the molybdopterin synthase activating enzyme in molybdenum cofactor (Moco) biosynthesis, is the best characterized protein from this family [10]. *E. coli* MoeB was shown to activate the small subunit of MPT synthase MoaD to form an acyl-adenylate intermediate at its C-terminal glycine [11]. Subsequently, the MoaD acyl-adenylate is converted to a thiocarboxylate either by a L-cysteine desulfurase using L-cysteine as sulfur source [12] or by a rhodanese-like protein containing a protein-bound sulfane sulfur [13]. In all eukaryotic and several bacterial MoeB homologs a rhodanese-like domain is fused to the C-terminus of MoeB (referred to as MoeBR and MoeZR) [9]. This rhodanese-like domain was shown to be directly involved in the generation of the thiocarboxylate group of the MoaD homolog in humans [13]. While all sequenced eukaryotic MoeB-homologs so far contain the C-terminal rhodanese-like domain, the bacterial homologs are much more divergent. Here, either a MoeB-homolog is present, a MoeB-homolog with a C-terminal rhodanese-like domain (MoeBR), a MoeZ-homolog, a MoeZ-homolog with a C-terminal rhodanese-like domain (MoeZR) or a combination of several of these proteins [9]. MoeBR and MoeZR proteins share a high amino acid sequence identity. The main difference between both proteins are two conserved CXXC motifs near the C-terminus of the MoeB-domain. While these two motifs are present in all MoeB and MoeBR proteins, MoeZ and MoeZR homologs are mostly missing the second motif completely, while the first motif comprises the consensus sequence NYRD [9].

Recently, the role of genes involved in Moco biosynthesis were described for *M. tuberculosis* [14]. While *E. coli* contains single copies of the genes for Moco biosynthesis, a distinguishing feature of members of the *M. tuberculosis* complex is their possession of multiple homologs associated with the steps of the Moco biosynthesis pathway for the conversion of GTP to cyclic pyranopterin monophosphate (cPMP), the formation of molybdopterin (MPT) by insertion of two sulfur atoms into cPMP and the final insertion of molybdate to the dithiolene sulfurs of MPT, thus forming Moco [14], [15]. According to our previous nomenclature we refer to the protein for the *moeB2* gene as MoeBR and for the protein to the *moeB1* gene as MoeZR. A gene region containing *moaA1-moaB1-moaC1-moaD1* and *moeB2* was acquired by horizontal gene transfer in *M. tuberculosis* H37Rv and *M. bovis* BCG [14]. In addition, a *moaX* gene was identified located downstream of this cluster which comprises a fusion of *moaD* and *moaE* and was shown to form active components of MPT synthase, since the *moaX* gene was able to complement both, a *M. smegmatis moaD2* and *moaE2* mutant [14].

Previously, the effects of several *M. tuberculosis* mutants in genes for Moco biosynthesis were described [14]: a *M. tuberculosis moeB1* mutant is defective in arresting phagosome maturation [16]; *moaC1* and *moaX* mutants showed a reduced ability to parasitize macrophages [17]; and a *moaC1* mutant was attenuated for growth in primate lungs [18]. In addition, the gene expression profile of *M. tuberculosis* in mice specifically identified *moaB2* as part of a gene cluster which was highly expressed *in vivo* [19]. Most recently, independent transposon mutants in *moaC1* and *moaD1* in the *M. tuberculosis* W-Beijing strain, GC1237, were identified by high-content, phenotypic cell-based screening as defective in the ability to arrest phagosome maturation [20]. An important role for nitrate reductase under anaerobic growth conditions has been ascribed for *M. tuberculosis* [14].

In this study we investigated the role of MoeBR and MoeZR in Moco biosynthesis. *Mycobacterial* MoeBR and MoeZR were purified and characterized in their activities. Both MoeBR and MoeZR showed thiosulfate∶sulfurtransferase activity. After coexpression of MoeZR and MoeBR with either MoaD1 or MoaD2 in *E. coli*, sulfurated MoaD-homologs for Moco biosynthesis were obtained. The sulfur-transfer was verified by mass spectrometry. Direct interaction between MoeZR, MoeBR and MoaD1, MoaD2 or CysO were analyzed by copurification studies. Our results show that both MoeBR and MoeZR have a role in Moco biosynthesis. While MoeBR preferentially interacts with both MoaD proteins, MoeZR has a dual role in the cell and is able to transfer the sulfur to either of the two MoaDs or CysO, a protein involved in cysteine biosynthesis.

## Results

### Test of functional complementation of *E. coli moeB^−^* cells with MoeBR and MoeZR

To analyze the role of the duplicated *E. coli* MoeB homologs from *M. tuberculosis* MoeBR and MoeZR in Moco biosynthesis, functional complementation studies of an *E. coli moeB^−^* strain were performed. To investigate the functional properties of both proteins, *M. tuberculosis* MoeBR and MoeZR were cloned into expression vectors (obtained from J. Kuper, EMBL Hamburg), resulting into fusion proteins with an N-terminal MBP-tag. For functional complementation studies, the corresponding vectors were expressed in the *E. coli moeB* mutant strain (plus 20 µM IPTG). Complementation of the strains by the *Mycobacterial* proteins would result in the production of active nitrate reductase, a Moco-containing enzyme, the activity of which is dependent on the ability of cells to synthesize Moco. The activity of nitrate reductase can be quantified in crude cell extracts (Table 1). The values in Table 1 show that MoeBR but not MoeZR alone was able to complement the *E. coli moeB* ^−^ strain at least to some extent (6-fold increase in comparison to the background), thus, it is speculated that MoeZR is unable to interact with the *E. coli* MoaD protein. To circumvent this, a coexpression with the corresponding *Mycobacterium* homolog would be necessary. For this purpose, we cloned the genes *moaD1*, *moaD2*, *moaX* and additionally cysO from *M. bovis* total DNA, which is commercially available. The amino acid sequences are identical with the ones of the *M. tuberculosis* proteins, so the *M. bovis* sequence should be suitable for protein expression and interaction studies of the purified proteins with *M. tuberculosis* MoeBR and MoeZR. MoaX is a fusion protein of MoaD and MoaE and was shown before to harbor MPT synthase activity [14]. The genes were cloned into P15A origin based expression vectors which allowed simultaneous replication of two vectors after transformation in the *E. coli moeB^−^* cells. The results in Table 1 show that in combination with MoaD1 and MoaD2, MoeBR and MoeZR were both able to functionally complement the *E. coli moeB^−^* strain. The determined nitrate reductase activities for MoeZR were 13–14 fold higher in conjunction with MoaD1 and MoaD2 in comparison to the background, which implies that MoeZR is well able to activate the *Mycobacterial* MoaD homologs but not MoaD from *E. coli*. The nitrate reductase activities for the complemented cells with MoeBR in addition to MoaD1 and MoaD2 were 1.9–2.3 increased in comparison to the single complementation with MoeBR, showing that MoeBR better interacts with its own partners. The nitrate reductase activities of MoeZR and MoeBR in conjunction with the MoaD homologs were comparable (Table 1). No functional complementation was obtained by coexpressing MoaX.

**Table 1:** Nitrate reductase activity after functional complementation of an *E. coli moeB^−^* strain with the *Mycobacterial* proteins MoeZR, MoeBR, MoaD1, MoaD2, and CysO.

MoeB-homolog^a^	MoaD-homolog^a^	Activity [U/OD_600_]^b^
*moeB^−^*	-	0.003±0.001
MoeB *E. coli*	-	0.233±0.033
-	MoaD *E. coli*	0.003±0.006
-	MoaD1	0.011±0.001
-	MoaD2	0.013±0.003
-	CysO	0.011±0.001
-	MoaX	0.012±0.001
MoeBR	-	0.020±0.005 **^c^^,^ d
MoeBR	MoaD1	0.046±0.009 **^d^
MoeBR	MoaD2	0.038±0.007 **^d^
MoeBR	CysO	0.013±0.002
MoeBR	MoaX	0.009±0.001
MoeZR	-	0.003±0.006
MoeZR	MoaD1	0.039±0.007 **^e^
MoeZR	MoaD2	0.042±0.008 **^e^
MoeZR	CysO	0.010±0.004
MoeZR	MoaX	0.012±0.005

a MBP-MoeBR and MBP-MoeZR were coexpressed in an *E. coli moeB^−^* strain together with His_6_-MoaD1, His_6_-MoaD2, His_6_-MoaX or His_6_-CysO as indicated.

b Nitrate reductase activity was determined in crude cell extracts by a spectroscopic assay using benzyl viologen as described by Jones and Garland [27]. One unit of nitrate reductase activity is described as the production of 1 µmol of nitrite per min per O.D. 600. Assays were performed in triplicate.

Significant differences at a level of *P*<0.01 (Student's *t* test) are indicated as **. All values represent means ± standard deviation (SD) for n = 9.

c significant difference of the MoeBR complemented strain compared to the *E. coli moeB^−^* strain.

d significant differences of the MoeBR/MoaD1 and MoeBR/MoaD2 complemented strains to the MoeBR complemented *E. coli* strain.

e significant differences of the MoeZR/MoaD1 and MoeZR/MoaD2 complemented strains to the MoeZR complemented *E. coli* strain.

### Purification of MoeBR and MoeZR

**Figure 1:**
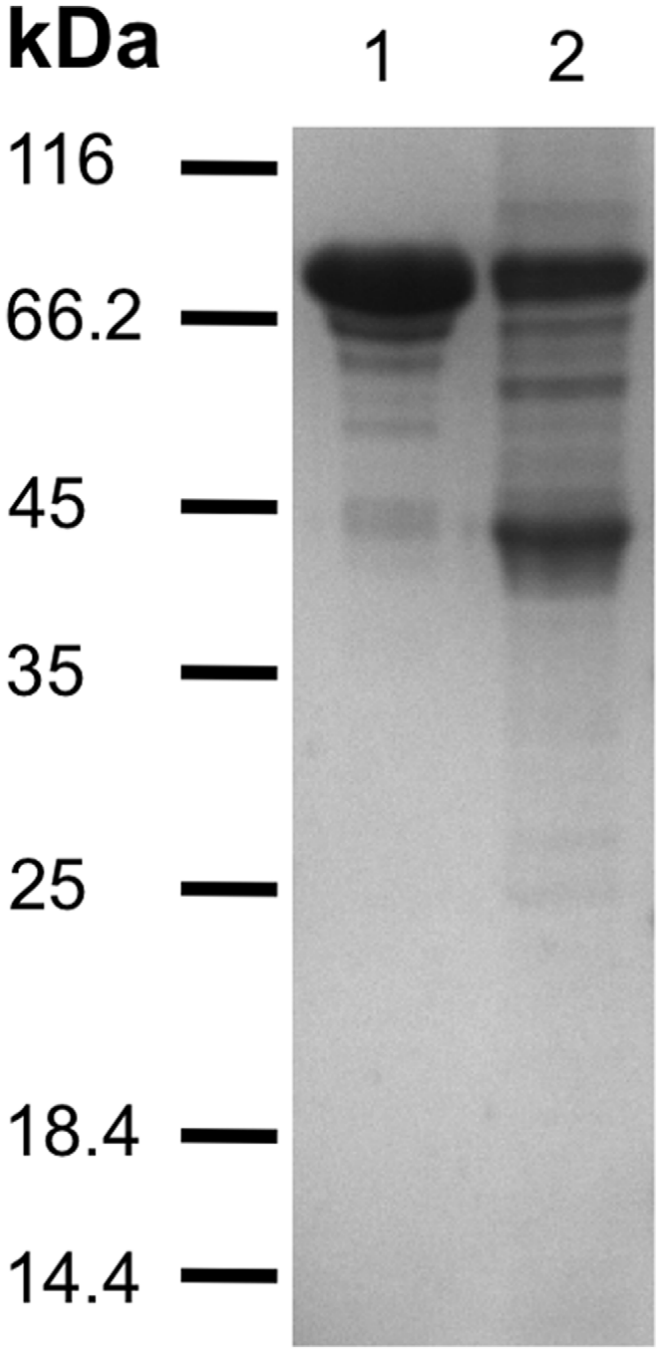
Purification of *M. tuberculosis* MoeZR and MoeBR after heterologous expression in *E. coli*. 5 µg of the purified proteins were separated by 15% SDS-PAGE and stained with Coomassie Blue. 1: MBP-MoeZR, 2: MBP-MoeBR. The band at 45 kDa in the MoeBR sample was shown to be MBP.

**Figure 2:**
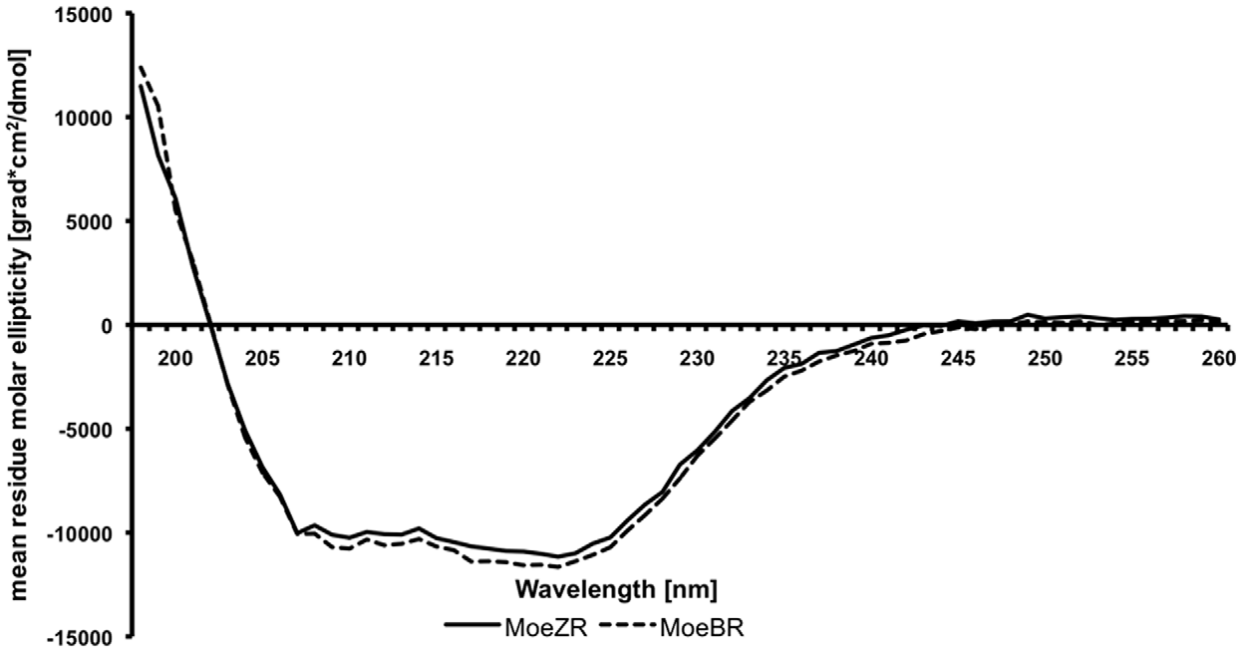
Circular Dichroism (CD) spectroscopy of MoeBR and MoeZR. CD on purified MBP-MoeBR and MBP_MoeZR were recorded in a 0.1 cm path-length Suprasil quartz cell with a Jasco-J715 CD spectrometer. Far-UV CD was recorded from 260-198 nm using a step size of 1 nm with a signal averaging time of 4 seconds at each wavelength step. 200 µL of MoeBR and MoeZR at a concentration of 0.1 mg/mL was used.

To further characterize MoeBR and MoeZR, both proteins were purified after heterologous expression in *E. coli*. For purification of MoeZR and MoeBR, both genes cloned in the pMalC2x vector were used. Expression of the proteins in BL21(DE3) cells resulted in N-terminal MBP-tagged recombinant proteins. MoeZR and MoeBR were purified by amylose affinity chromatography. One major band was visible on SDS-polyacrylamide gels with a size corresponding closely to the calculated molecular masses of the MBP-MoeZR fusion protein of 94 kDa and MBP-MoeBR with a mass of 93 kDa (Figure 1). However, some degradation products are visible on the SDS-polyacrylamide gel. The band at 45 kDa was determined to be MBP, as verified by MALDI peptide mapping. MoeZR and MoeBR were purified with a yield of 4.4 mg/L or 7.8 mg/L *E. coli* cells. To determine whether structural differences between MoeBR and MoeZR exist, CD spectra of both proteins were recorded. As shown in Fig. 2 the far-ultraviolet CD spectra revealed mainly no differences in the average composition of secondary-structural elements of MoeZR and MoeBR.

### Analysis of the sulfurtransferase activity of MoeZR and MoeBR

For MoeB-like proteins with a C-terminal rhodanese-like domain, it was shown before that they contain a conserved cysteine residue, which is part of a highly conserved six-amino acid active-site loop, that is essential for thiosulfate sulfurtransferase activity by formation of a persulfide group during catalysis. After purification of MoeBR and MoeZR, it was of interest to compare the activities of the C-terminal rhodanese-like domain of the purified proteins. *In vitro*, thiosulfate sulfurtransferase activity was measured by the method described by Sörbo [21]. The data in Table 2 show that MoeBR and MoeZR express thiosulfate sulfurtransferase activity. The k_cat_ and K_M_ values for MoeZR and MoeBR with thiosulfate are comparable as revealed by the k_cat_/K_M_ values, however, the k_cat_/K_M_ with cyanide is only half for MoeBR in comparison to MoeZR. In total, the k_cat_ values are 112–196 times higher in comparison to human MOCS3 [9] and comparable with yeast Uba4 [22], homologous proteins from eukaryotes.

**Table 2:** Kinetic parameters of the thiosulfate∶cyanide sulfurtransferase activity of purified MoeBR and MoeZR.

		Na_2_S_2_O_3_ a		NaCN^a^	
	k_cat_ [s^−1^]	K_M_ [mM]	k_cat_/K_M_ [s^−1^*mM^−1^]	K_M_ [mM]	k_cat_/K_M_ [s^−1^*mM^−1^]
MoeZR	2.25±0.20	1.67±0.28	1.35	0.81±0.07	2.78
MoeBR	3.92±0.23	4.19±0.42	0.94	3.28±0.12	1.20

a The Thiosulfate∶cyanide sulfurtransferase activity of MBP-MoeZR and MBP-MoeBR were measured by the colorimetric assay after Sörbo [21]. K_M_ and k_cat_ values were determined by varying concentrations of sodium thiosulfate (0.5–60 mM) and potassium cyanide (0.1–40 mM) with 0.5–1 µM enzyme.

### Copurification of MoeBR and MoeZR with MoaD1, MoaD2 and CysO

**Figure 3:**
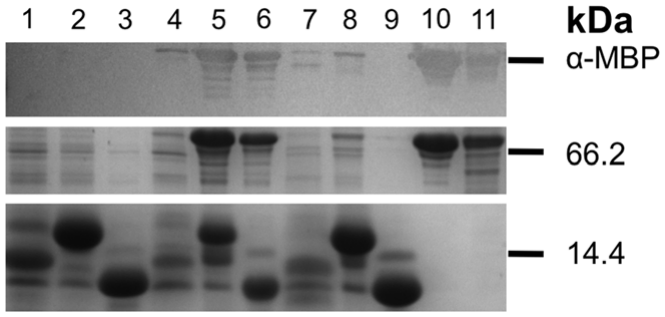
Copurification of MoeBR or MoeZR with MoaD1, MoaD2 and CysO. MBP-MoeBR and MBP-MoeZR were coexpressed in an *E. coli moeB^−^* strain together with His_6_-MoaD1, His_6_-MoaD2 or His_6_-CysO and purified by Ni-NTA chromatography afterwards. As control, His_6_-MoaD1, His_6_-MoaD2 or His_6_-CysO were also expressed without MoeBR and MoeZR in the *E. coli moeB^−^* strain. The proteins were separated by 15% SDS-PAGE and stained with Coomassie Blue or, in addition, the presence of MBP-MoeBR and MBP-MoeZR was determined by immunodetection using polyclonal MBP antisera (Sigma). 1: His_6_-MoaD1, 2: His_6_-MoaD2, 3: His_6_-CysO, 4: His_6_-MoaD1/MBP-MoeZR, 5: His_6_-MoaD2/MBP-MoeZR, 6: His_6_-CysO/MBP-MoeZR, 7: His_6_-MoaD1/MBP-MoeBR, 8: His_6_-MoaD2/MBP-MoeBR, 9: His_6_-CysO/MBP-MoeBR.

To additionally confirm that MoeZR interacts with MoaD1 and MoaD2, it should be possible to copurify the protein complexes after coexpression in *E. coli*. Plasmids expressing His_6_-MoaD1, His_6_-MoaD2, and His_6_-CysO were cotransformed with MBP-MoeZR and MBP-MoeBR and subjected to Ni-NTA affinity chromatography after coexpression. The results in Figure 3 show, that with His_6_-MoaD1, His_6_-MoaD2 and His_6_-CysO, MBP-MoeZ was copurified. In contrast, MBP-MoeBR was only copurified in the mixtures containing His_6_-MoaD1 and His_6_-MoaD2. Thus, MoeBR does not interact with CysO. The results were additionally confirmed by immunoblot analyses using an MBP-antibody (Figure 3) and in addition by MALDI peptide mapping (data not shown). Both analyses confirmed clearly, that MoeBR does not interact with CysO.

### Electrospray Mass Spectrometry of MoaD1, MoaD2 and CysO after coexpression with MoeBR and MoeZR

**Figure 4:**
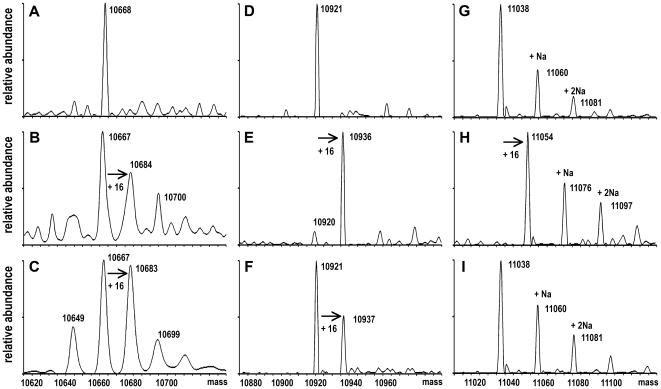
Deconvoluted ESI mass spectra of MoaD1, MoaD2, and CysO. The sulfuration level of His_6_-MoaD1 (A–C), His_6_-MoaD2 (D–F) and His_6_-CysO (G–I) was analyzed after expression in *E. coli moeB^−^* strain either alone or after coexpression with MBP-MoeZR or MBP-MoeBR. After coexpression, His_6_-MoaD1, His_6_-MoaD2 and His_6_-CysO were purified by Ni-NTA chromatography and subjected to ESI-MS. The mass increase of +16 Da corresponds to the exchange of an oxygen versus a sulfur atom to generate thiocarboxylated proteins. (A) His_6_-MoaD1, (B) His_6_-MoaD1 after coexpression with MBP-MoeZ, (C) His_6_-MoaD1 after coexpression with MBP-MoeBR. (D) His_6_-MoaD2, (E) His_6_-MoaD2 after coexpression with MBP-MoeZR, (F) His_6_-MoaD2 after coexpression with MBP-MoeBR. (G) His_6_-CysO, (H) His_6_-CysO after coexpression with MBP-MoeZR, (I) His_6_-CysO after coexpression with MBP-MoeBR. Additional peaks with mass increments of 22 Da are due to the corresponding Na-salts of proteins.

To directly prove the existence of a thiocarboxylate group at the C-terminal glycine of either His_6_-MoaD1, His_6_-MoaD2 or His_6_-CysO after coexpression with MBP-MoeBR or MBP-MoeZR, respectively, the purified proteins were subjected to electrospray ionization (ESI) mass spectrometry, which allows the detection of the oxygen versus sulfur exchange due to its characteristic mass shift of 16 Da. As shown in Fig. 4, His_6_-MoaD1 and His_6_-MoaD2 contained a C-terminal thiocarboxylate group after coexpression with MBP-MoeBR and MBP-MoeZR. In contrast, on His_6_-CysO a thiocarboxylate group was only determined when MoeZR was present for coexpression. The presence of the modification at the C-terminal glycine was verified by MS/MS based on differences after carboxyterminal fragmentation analyses (data not shown). This clearly shows that both MoeBR and MoeZR are able to activate MoaD1 and MoaD2, however, only MoeZR is able to form a stable complex with CysO which leads to the formation of the C-terminal thiocarboxylate group on CysO.

## Discussion

**Figure 5:**
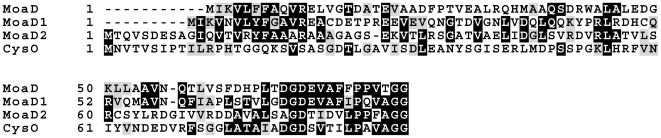
Amino acid sequence comparison of *E. coli* MoaD, *M. tuberculosis* MoaD1, *M. tuberculosis* MoaD2, and *M. tuberculosis* CysO. Identical amino acids are boxed in black and homologous amino acids residues are shaded in grey.

Our studies involve the characterization of *Mycobacterial* MoeZR and MoeBR. A distinguishing feature of members of the *M. tuberculosis* species is their possession of multiple homologs associated with the first step of Moco biosynthesis [14], [23]. Two homologs of the *E. coli* MoeB protein were identified in *M. tuberculosis* H37Rv and *M. bovis* BCG Pasteur (Table 3). The corresponding genes were annotated as *moeB1* and *moeB2* [*14*]. According to our previous nomenclature we refer to the protein for the *moeB2* gene as MoeBR and for the protein to the *moeB1* gene as MoeZR, since both proteins contain a C-terminal rhodanese-like domain, which is present in some bacterial and eukaryotic homologs including human MOCS3, but not in *E. coli* MoeB [9]. For *M. tuberculosis* MoeZR it was described that it plays a role in a novel cysteine biosynthesis pathway [2]. This cysteine biosynthesis pathway involves a small sulfur carrier protein, CysO, which carries a C-terminal thiocarboxylate group and a cysteine synthase, CysM [4]. CysM reacts with O-phosphoserine to form an α-aminoacrylate intermediate [7]. The sulfur is then provided by CysO and cleavage of cysteine results in the release of carboxylated CysO [3]. For the regeneration of CysO thiocarboxylate, it has been suggested that MoeZR first activates CysO by acyl-adenylation of the C-terminus and then the thiocarboxylate group is formed by involvement of a sulfane sulfur bound to the C-terminal rhodanese-like domain of MoeZR [2]. The sulfur source so far remains unknown. These studies were performed *in vitro* by using crude cell extracts, *in vivo* evidence for the involvement of MoeZR in CysO activation was not shown to date. It was only reported, that the expression of MoeZR is upregulated under the same conditions that produce upregulation of CysO and CysM expression, mainly conditions of oxidative stress [3]. Our studies performed in *E. coli* give direct evidence, that CysO specifically interacts with MoeZR and that a thiocarboxylate group is formed on CysO only in the presence of MoeZR. However, our studies also show that MoeZR has a dual role in *Mycobacteria* and is also involved in Moco biosynthesis. Functional complementation studies of an *E. coli moeB* mutant showed that MoeZR was able to complement the *E. coli* MoeB function only in the presence of either MoaD1 or MoaD2. This shows that MoeZR does not interact with *E. coli* MoaD. The amino acid sequence alignment of MoaD1, MoaD2, CysO and *E. coli* MoaD shows, that MoaD2 is more related to CysO than to MoaD1 (Fig. 5). Also, MoaD1 shares higher amino sequence identities to *E. coli* MoaD than to MoaD2. Thus, we speculate that *in vivo* in *M. tuberculosis* MoaD1 might preferentially interact with MoeBR, a speculation supported by the closer amino acid sequence identities to the *E. coli* congeners and the fact that both genes were simultaneously acquired by horizontal gene transfer. However, our studies also show that MoeBR is also able to interact with MoaD2. On the other hand, MoaD2 might be the more specific interaction partner to MoeZR, a speculation based on the fact that some *Mycobacteria* strains only contain MoeZR and MoaD2 and no additional MoaD1 copies. Our study clearly shows that MoeZR has a role in Moco biosynthesis in *Mycobacteria* in addition to its role in cysteine biosynthesis. Our results are also supported by the fact that the *M. avium*, *M. smegmatis*, *M marinum*, and *M. ulcerans* strains only have a MoeZR homolog and no additional MoeB-homolog (Table 3), while expressing active molybdoenzymes, as shown for *M. smegmatis* before [14]. Thus, even though MoeZR is more distantly related to *E. coli* MoeB, it is the more widely distributed MoeB-homolog in *Mycobacteria*. Since MoeZR can perform two roles in Moco biosynthesis and cysteine biosynthesis, this might have been more advantageous to *Mycobacteria* than keeping two MoeB-homologs. Our studies clearly revealed, that the role of MoeBR is restricted to Moco biosynthesis. MoeBR is unable to interact with CysO, however, it is able to interact with both MoaD1 and MoaD2. Since the *Mycobacterial* MoeZR and MoeBR proteins showed a higher ability to complement the *E. coli* MoeB function when their own MoaD congeners were present. The main difference between the *E. coli* and the *Mycobacterial* MoeB-like proteins is that both *Mycobacterial* congeners contain a C-terminal rhodanese-like domain. Thus, a fusion of the rhodanese-like protein to the MoeB-domain was sustained during evolution in *Mycobacteria*. In contrast in *E. coli*, IscS was shown to be the primary sulfur donor for Moco biosynthesis, transferring the sulfur to MoaD [12]. Recently, a separate rhodanese-like protein was identified in *E. coli*, named YnjE, which was shown to act as a mediator between the sulfurtransfer from IscS to the MoaD/MoeB complex in *E. coli*, making the interaction more specific [24]. Thus, in *E. coli* the sulfur transfer pathway is realized by several protein partners, and not by fusion proteins. The primary sulfur donor for Moco biosynthesis in *Mycobacteria* remains to be elucidated. However, IscS homologs were identified [25] (Table 3), which might act as the primary sulfur donor to the rhodanese-like domain, as shown for the human MOCS3 protein [26].

**Table 3:** Distribution of genes involved in Moco and cysteine biosynthesis in different *Mycobacterium* strains.

	*M. tuberculosis* H37Rv	*M. bovis* BCG Pasteur	*M. smegmatis* mc2155	*M. avium* 104	*M. marinum* M	*M. ulcerans* Agy99
*moaD1*	RV3112	BCG_3137	-	-	-	-
*moaD2*	Rv0868c	BCG_0920c	MSMEG_5699	MAV_0997	MMAR_4664	MUL_0282
*cysO*	Rv1335	BCG_1397	MSMEG_4906	-	MMAR_4063	MUL_3927
*moaE1*	Rv3119	-	-	-	-	-
*moaE2*	Rv0866	BCG_0918	MSMEG_5701	MAV_0995	MMAR_4666	MUL_0284
*moaX*	Rv3323c	BCG_3389c	-	-	-	-
*moeZR*	Rv3206c	BCG_3232c	MSMEG_1937	MAV_4153	MMAR_1352	MUL_2527
*moeBR*	Rv3116	BCG_3141	-	-	-	-
*iscS*	Rv3025c	BCG_3048c	MSMEG_2357	MAV_3872	MMAR_1689	MUL_1927
*cysM*	Rv1336	BCG_1398	MSMEG_4905	MAV_0444	MMAR_4629	MUL_0242

Purification and characterization of MoeBR and MoeZR showed, that both proteins had the same secondary structure. MoeBR and MoeZR were purified in an active form which showed comparable thiosulfate∶cyanide sulfurtransferase activities, however, thiosulfate is most likely not the physiological sulfur source, since the K_m_ values were unphysiological. The k_cat_ values with thiosulfate were comparable to the ones identified for other MoeB-like proteins containing a C-terminal rhodanese-like domain, like the human yeast Uba4 [22].

We also planned to purify and characterize MoaX. MoaX is a fusion protein of MoaD and MoaE [14]. For the accurate function of MoaD, the C-terminal glycine has to be accessible for the adenylation and sulfurtransfer reaction by the MoeB-homolog and for the subsequent sulfurtransfer to cPMP in conjunction with MoaE. Williams et al. [14] showed that MoaX was able to complement both, an *M. smegmatis moaD2* mutant and a *moaE2* mutant, assuming that an active MPT synthase was produced. The assumption was that MoaX is cleaved in the cell and a separate and active MoaD protein is generated. Our studies show that MoaX was not able to complement the *E. coli moeB* mutant in conjunction with MoeBR or MoeZR, and in addition single *E. coli moaD* and *moaE* mutant strains were also not complemented by *moaX* (data not shown). Thus, in *E. coli* the system for cleavage of MoaX seems to be missing. Unfortunately, we did not succeed to express and purify MoaX for further characterization, since the majority of the protein was expressed in inclusion bodies (data not shown). So far, the role of MoaX for *Mycobacteria* remains unclear. Especially since only *M. tuberculosis* and *M. bovis* species have acquired a MoaX homolog. It also remains possible that MoaX has a role in the cell apart from Moco biosynthesis, however, this is only a speculation.

**Figure 6:**
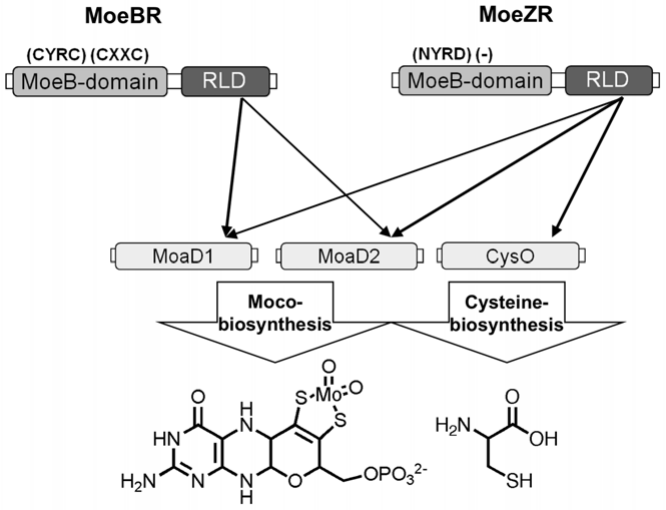
Proposed model for the role of MoeZR and MoeBR in *Mycobacteria*. Functional complementation studies of an *E. coli moeB^−^* strain showed that both MoeZR and MoeBR are able to interact with either MoaD1 or MoaD2. In addition, MoeZR is able to sulfurate CysO for cysteine biosynthesis, while MoeBR is not able to interact with CysO.

It is not clear yet, why different *Mycobacteria* strains contain several homologs of genes for Moco biosynthesis. The *moaA1-moaB1-moaC1-moaD1* cluster and *moeB2* (coding for MoeBR) were acquired by horizontal gene transfer in *M. tuberculosis* and *M. bovis* [14]. Also, the *moaA3-moaB3-moaC3-moaX* gene cluster was acquired by horizontal gene transfer in these strains. Williams et al. [14] showed that the *moaA1-moaD1* cluster is dispensable when grown with nitrate in the medium. Thus, the additional copies of the genes from Moco biosynthesis might have an advantage under special growth conditions, which are not necessary to maintain the general growth of *Mycobacteria*. Our studies show that both MoeBR and MoeZR are able to perform Moco biosynthesis. Since almost all *Mycobacterium* species have a MoeZR homolog, MoeZR seems to perform a dual role in the cell, the role in Moco biosynthesis in conjunction with MoaD2 and the role in cysteine biosynthesis in conjunction with CysO (Fig. 6). The role of MoeBR seems to be restricted to Moco biosynthesis and most likely it preferentially interacts with MoaD1 in the cell, since both genes have been aquired simultaneously by horizontal gene transfer. However, both MoeBR and MoeZR retained their ability to interact with both MoaD1 and MoaD2 (Fig. 6). How both processes, the biosynthesis of Moco and cysteine are regulated in the cell, has to be determined in future studies. Since L-cysteine is the likely sulfur donor for Moco biosynthesis and an L-cysteine desulfurase acts as sulfur donor to MoeZR, MoeZR might be the link for both biosynthetic pathways and could be the main switch for feedback regulation.

## Materials and Methods

### Bacterial Strains, Plasmids, Media, and Growth Conditions

The bacterial strains and plasmids used in this work are listed in Table 4. *E. coli* BL21(DE3) cells were used for expression of the *Mycobacterial* proteins. Bacterial cultures were generally grown in LB medium under aerobic conditions at 30°C. When required, 150 µg/mL ampicillin or 50 µg/mL chloramphenicol was added to the medium.

**Table 4:** Strains and Plasmids used in this study.

Species and strain or plasmid	Relevant characteristics	Source or reference
***E. coli***		
BL21(DE3)	F- *ompT hsdDB* (rB-mB-) *gal dcm* (DE3)	[28]
*moeB^−^(DE3)*	F- rpsL, MJ441 chlN (DE3)	[10]
**Plasmids**		
pMal_*MtmoeBR*	pMal_*MtmoeBR*, *Eco*RI/*Hin*dIII, resulting in a N-terminal MBP-tag	This study
pSM2	pMal_*MbmoeZR*, *Eco*RI/*Hin*dIII, resulting in a N-terminal MBP-tag	This study
pCO10	pACYCDuet1_*MbmoaD1*, *Bam*HI/*Not*I, resulting in a N-terminal His_6_-tag	This study
pCO13	pACYCDuet1_*MbmoaD2*, *Bam*HI/*Not*I, resulting in a N-terminal His_6_-tag	This study
pCO16	pACYCDuet1_*MbcysO*, *Bam*HI/*Not*I, resulting in a N-terminal His_6_-tag	This study
pSM1	pACYCDuet1_*MbmoaX*, *Bam*HI, resulting in a N-terminal His_6_-tag	This study
pMW15eB	pET15b-*EcmoeB*, *Nde*I/*Bam*HI	[10]
pMW15aD	pET15b-*EcmoaD*, *Nde*I/*Bam*HI	[6]

### Protein Expression and purification

The clones for the expression of MoeBR and MoeZR were obtained from the EMBL in Hamburg. For coexpression of *M. tuberculosis* MoeZR or MoeBR, and *M. bovis* MoaD1, MoaD2, MoaE2, or CysO, the corresponding plasmids (Table 4) were cotransformed into *E. coli* BL21(DE3) cells. For overexpression of the recombinant MBP-tagged MoeBR and MoeZR proteins, 1 liter of LB medium (for proteins on the pMalC2x vector, 0.2% glucose was added) was inoculated with 20 ml overnight culture of BL21(DE3) carrying the respective plasmid and cultivated at 37°C to an optical density at 600 nm of 0.6 before protein expression was induced by addition of 100 µM isopropyl-β-D-thiogalactopyranoside. Growth was continued for 4–6 h at 30°C, and cells were harvested by centrifugation and resuspended in 100 ml of 20 mM Tris, 200 mM NaCl, 1 mM EDTA, pH 7.4. Cell lysis was achieved by several passages through a cell disrupter (Constant systems).

After centrifugation (20,000× g, 20 min), the supernatant of MBP-tagged MoeBR and MoeZR was combined with 1–2 ml of amylose affinity matrix equilibrated with 20 mM Tris-HCl, 200 mM NaCl, 1 mM EDTA, 1 mM DTT, pH 7.4. After two washing steps with 12 column volumes of 20 mM Tris-HCl, 200 mM NaCl, 1 mM EDTA, 1 mM DTT, pH 7.4 the protein was eluted in 3 mL fractions with 20 mM Tris-HCl, 200 mM NaCl, 1 mM EDTA, 1 mM DTT, 10 mM maltose, pH 7.4. After purification all proteins were dialyzed into 100 mM Tris-HCl, 200 mM NaCl, pH 7.4 prior to use. MoeZR was further purified by size exclusion chromatography on a Superose 12 column equilibrated in 100 mM Tris, 200 mM NaCl, pH 7.4.

### Functional complementation of an *E. coli moeB* mutant strain

For functional complementation, the *E. coli moeB^−^* strain was transformed with the corresponding expression plasmids for *E. coli* MoeB (pMW15eB) and MoaD (pMW15aD), control, and the *Mycobacterium tuberculosis* MoeBR (pMal_MtMoeBR), MoeZR (pSM2) alone or for coexpression with *M. bovis* His_6_-MoaD1 (pCO10), His_6_-MoaD2 (pCO13), His_6_-CysO (pCO16), His_6_-MoaX (pSM1). For quantitative determination of nitrate reductase activity, the transformed *E. coli* cells were grown aerobically at 30°C in 5 ml of LB medium containing 15 mM nitrate. Protein expression was induced with 20 µM IPTG. Nitrate reductase activity in crude cell extracts was determined by a spectroscopic assay using benzyl viologen as described by Jones and Garland [27]. Nitrate reductase activity is related to the amount of cells measured at an O.D. at 600 nm. One unit of nitrate reductase activity is described as the production of 1 µmol of nitrite per min per O.D. 600.

### Thiosulfate∶cyanide sulfurtransferase activity

Thiosulfate∶cyanide sulfurtransferase activities of MoeZR and MoeBR were measured by the classic colorimetric method after Sörbo [21], which is based on the absorbance of the complex formed between ferric ion and thiocyanate at 460 nm. Reaction mixtures in 100 mM Tris-acetate (pH 8.6), contained varying concentrations of sodium thiosulfate (0.5–60 mM) and varying concentrations of potassium cyanide (0.1–40 mM) in a volume of 0.5 mL. Reactions were started by the addition of the enzyme in a range of 0.5–1 µM, depending on the MoeB-like variant used. After an incubation time of 0.5–1 min at 25°C, formaldehyde (15%, 250 µL) was added to quench the reaction. Color was developed by the addition of 750 µL of ferric nitrate reagent [100 g of Fe(NO_3_)_3_×9H_2_O and 200 mL of 65% HNO_3_ per 1500 mL]. After a further incubation for 10 min, thiocyanate (complexed with iron) was quantified at 460 nm using ε = 4200 M^−1^×cm^−1^.

### Electrospray Ionization MS

Aliquots (1–3 μL) of MoaD1, MoaD2, CysO purified from extracts coexpressed with MoeBR or MoeZR dissolved in 5 mM NH_4_OAc buffer were diluted 1∶1 with methanol followed by addition of 1–10% formic acid (final protein concentration 1–10 pmol/µL) and applied to gold-coated nanospray glass capillaries, which were placed orthogonally in front of the entrance hole of a Q-TOFmicro instrument (Micromass, Manchester, U.K.). A voltage of approximately 1000 V was applied to the capillary, and ions were separated by the time-of-flight analyzer of the mass spectrometer. Protein spectra were deconvoluted using the MaxEnt1 software package (Micromass, Manchester, UK).

### CD spectroscopy

Far-UV CD spectra of 0.1 mg/mL enzyme samples were recorded in 100 mM Tris, 200 mM NaCl, pH 7.4 using a Jasco J-715 CD-spectrophotometer. The scanning mode was set step-wise, each nm a data pitch was recorded, the response time was 4 seconds and each measurement was repeated 3 times.
